# Context Is Key: Comparative Biology Illuminates the Vertebrate Microbiome

**DOI:** 10.1128/mBio.00153-20

**Published:** 2020-03-10

**Authors:** Sarah M. Hird

**Affiliations:** a Department of Molecular and Cell Biology, University of Connecticut, Storrs, Connecticut, USA; b Institute for Systems Genomics, University of Connecticut, Storrs, Connecticut, USA

**Keywords:** birds, comparative studies, mammals, microbiome, microbiota

## Abstract

Microbes affect vertebrates on timescales from daily to evolutionary, and the cumulative effect of these interactions is immense. However, how microbiomes compare across (host) species is poorly understood, as most studies focus on relatively few species. A recent mBio article by S. J. Song, J. G. Sanders, F. Delsuc, J. Metcalf, et al. (mBio 11:e02901-19, 2019, https://doi.org/10.1128/mBio.02901-19) expands our collective understanding of the vertebrate microbiome by analyzing ∼900 species. They demonstrate that patterns within mammals contrast with those within birds. Their results suggest many hypotheses about the role of host ecology and evolution on microbiome variation. Bats, the only volant mammals, appear to contradict many of the general mammal microbiome trends, in some ways resembling birds. What role has powered flight, and the evolution thereof, played in microbiome structure and function? Comparative methods, mechanistic hypotheses, and theory will elucidate this exciting question (and others) that we can ask using Song, Sanders et al.’s data and results.

## Abstract (precis)

Microbes affect vertebrates on timescales from daily to evolutionary, and the cumulative effect of these interactions is immense. However, how microbiomes compare across (host) species is poorly understood, as most studies focus on relatively few species. A recent mBio article by S. J. Song, J. G. Sanders, F. Delsuc, J. Metcalf, et al. (mBio 11:e02901-19, 2019, https://doi.org/10.1128/mBio.02901-19) expands our collective understanding of the vertebrate microbiome by analyzing ∼900 species.

## COMMENTARY

Microbes affect vertebrate biology on many axes and scales. While we know a great deal about the microbiomes of a few species, most of the ∼65,000 vertebrate species have microbiomes unknown to science. To understand biology requires a broad view of biodiversity. With too narrow a view, we risk thinking a conclusion describes a general principle when its application is actually quite limited. Comparative studies can both address specific questions and provide context; a well-sampled distribution can quantify “normal” and detect outliers. In a recent mBio article, Song, Sanders et al. (1) collected and analyzed thousands of host-associated microbiome samples from almost 900 vertebrate species. This data set represents a significant first—an effort from dozens of individuals and institutions across the globe to collect microbiome samples from more than 1% of vertebrate species. Thus, a new era of microbiome analysis is upon us; what can we learn when microbiomes are presented in the context of their near and distant evolutionary relatives?

One of the strongest results of Song, Sanders et al. is that mammal microbiomes may be the exception rather than the rule regarding the relationship between hosts and microbiomes. Song, Sanders et al. find that diet and phylogeny explain a nontrivial (*R*^2^ = 17%) and significant amount of mammal microbiome variation—a finding consistent with many previous studies. Bird phylogeny explains a small (*R*^2^ = 1.7%) but significant (*P* < 0.001) amount of the avian microbiome variation, consistent with the low end of previous studies, and this value is similar to the nonavian reptiles and amphibians in the data set (*R*^2^ ∼ 3%). Phylosymbiosis (the concurrent branching pattern between a host phylogeny and microbiome similarity; see reference 2) is widespread within mammals, whereas the pattern is weak or absent in amphibians, lizards, turtles, and birds. Consistent with this, the microbes within mammalian microbiomes are often taxonomically restricted, exhibiting high host specificity. Birds show a dissimilar pattern of low specificity (specificity could not be tested in the other vertebrate clades). The authors note that there is growing evidence that questions whether findings from mammals generalize across vertebrates; Song, Sanders et al. add to this evidence.

The many adaptations of mammals provide hypotheses for mammalian microbiome exceptionalism. Their namesake adaptation, mammary glands, produce milk, which constitutes a direct path for vertical inheritance of (portions of) the microbiome. Additional mammalian traits, such as endothermy, live birth, herbivory, and large sizes, likely also influence the microbiome. All that being said, the degree that host class explains microbiome variation was surprisingly low—only 4.9%—which indicates that variation in the microbiomes of vertebrates is largely contained within classes and not distributed among them.

Another major result from Song, Sanders et al. is that the exception to mammalian exceptionalism appears to be bats. Bat microbiomes are more bird-like than mammal-like in their degree of phylosymbiosis, taxonomic composition, and specificity of the microbiome, and the two groups overlap nearly entirely in the ordination containing all the samples. This is a remarkable finding that the authors suggest may be due to both lineages using powered flight for locomotion.

In their concluding remarks, the authors say that comparative methods offer a way to discover and validate mechanisms responsible for the patterns in their data. Looking forward, Song, Sanders et al. should spur development in three key areas of microbiome research:

• Application of phylogenetic comparative methods to microbiome data
• Development and testing of mechanistic hypotheses to dissect and validate the discovered associations
• Development and testing of protocols for consistent sampling of microbiomes from diverse species.

## 

### Phylogenetic comparative methods.

Comparative methods detect patterns and processes shared across multiple species. Furthermore, all organisms are related by phylogeny, and many traits are not independent of that relationship, so phylogenetic comparative methods (PCMs) are needed to account for that relationship (3). PCMs can analyze the evolution of traits in a phylogenetic framework and estimate ancestral states. If we are interested in the relationship between microbiome traits and host life history traits, we need to look at them in a phylogenetic context. Powered flight has evolved four times—twice in extant vertebrates (birds and bats), once in insects, and once in extinct pterosaurs. The evolution of powered flight is difficult to study, as the fossil record is both patchy and incomplete. Studying how the evolution of flight affected the microbiome is even more difficult because we entirely lack a fossil record of the microbiomes that existed in the preflight lineages. Since major evolutionary transitions tend not to be replicated, the power of comparative methods to resolve their causes and consequences is fundamentally limited (4). Fortunately, there are many underlying mechanistic hypotheses that we can robustly test in field and laboratory settings.

### Mechanistic hypothesis testing.

**FIG 1:**
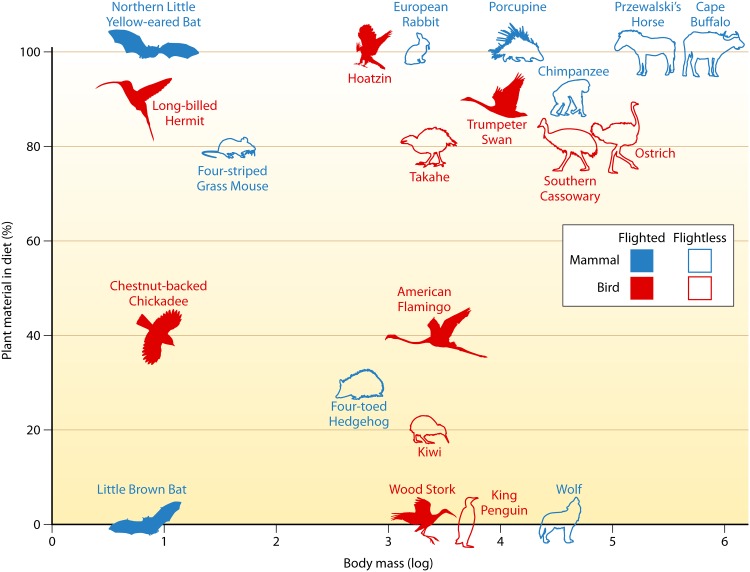
Whereas flight (filled shapes) has only evolved once in birds (in red) and once in mammals (in blue), both classes span orders of magnitude in size and contain species with diets that range from 0 to 100% plant material. These traits may be useful for mechanistic hypothesis generation and testing regarding microbiome diversity. (Data from Song, Sanders et al., Data File 1.)

What aspects of host biology are causing the patterns detected by Song, Sanders et al.? Several mechanistic hypotheses are discussed in Song, Sanders et al. that are fertile ground for testable experiments across clades. While flight has only evolved once in each group, birds and mammals share considerable variation in many pertinent continuous traits, such as size and diet (Fig. 1).

One such hypothesis is that birds and bats share several physical adaptations of the gut that may influence the microbiome, including shorter intestinal length and more rapid digestion times than their nonflying relatives. Rate of digestion is influenced by many aspects of host physiology and may be a significant contributor to microbiome variation in terms of diversity, composition, stability, specificity, and strength of signal in the data (i.e., error). The mean retention time (MRT) of food could impact how degraded the diet-associated microbes are upon excretion from the host (increasing the noise/signal ratio). MRT could impact how strongly resident microbes adhere to mucosal linings (again, increasing the noise/signal ratio). MRT dictates how much time resident microbes have to multiply on digesta. MRT is related to how frequently an organism excretes, which brings up additional hypotheses on the comparability of fecal samples (“Are all poops created equal in terms of sampling effort?”). Retention time can be measured and experiments can be designed to quantify MRT’s effects on the microbiome. Comparison across clades with similar gut retention times despite differences in overall size and ecology will elucidate which aspects of the microbiome are affected by this property. MRT is related to the diet and size of a host, making it well suited to phylogenetic comparative methods.

There are many testable hypotheses that, in the proper phylogenetic framework, may or may not implicate flight as an ultimate source of microbiome variation. Going forward and building off the results of Song, Sanders et al., we must construct mechanistic hypotheses for how adapted structures, unique ecologies, and interactions between host and environment influence specific features of the microbiome. Functional validation of the hypotheses in the lab and the development and use of theory are other ways to test what processes are important in a comparative framework.

### Consistent sampling.

A last lesson learned from Song, Sanders et al. concerns sample collection. The logistics of collecting samples from thousands of individuals are complex and constitute much of the reason that providing open access to the data is so beneficial to the scientific community. In a lab setting, best practices may be that a single individual collects all the samples for a study to minimize inconsistencies across collections. It is unreasonable to apply this standard to large comparative studies, and yet, sampling consistency is still paramount. Controlling for the various human and location-linked variables that could influence the microbiome is critical. Sample storage methods accounted for nearly as much variation as host class (bird versus mammal) in the data set (*R*^2^ = 4.6% versus 4.9%). There are currently few ways to account for such a bias after it has been introduced, and the easiest way to avoid wasted effort or confounded results is to be as consistent as possible with all samples, from sampling design through to data analysis. Of course, this is not always feasible; in remote locations, immediately freezing samples may be impossible (for example), and historical sample storage methods cannot be “reversed.” Going forward, we microbiome scientists should standardize collection methods as much as possible and account for and report effects that have been introduced.

We will likely also have to grapple with “microvariation” of microbiomes across a single host and a single sample type. Song, Sanders et al. report a minimal effect of captivity on the overall microbiome variation but also note that more of the birds and bats in the data set were sampled in captivity than nonbat mammals. Captivity influences the microbiome in many taxa, and this is likely due to the multidimensional ways captivity differs from natural conditions (5). Could captivity alter volant vertebrate microbiomes more than it does for their nonflying relatives?

The 30,000-foot view offered by Song, Sanders et al. provides the field with much to consider and exciting hypotheses to test. Microbiome science has much to gain from comparative biology now that such data are within reach.
